# Association between Urinary Advanced Glycation End Products and Subclinical Inflammation in Children and Adolescents: Results from the Italian I.Family Cohort

**DOI:** 10.3390/nu14194135

**Published:** 2022-10-05

**Authors:** Margherita Borriello, Fabio Lauria, Ivana Sirangelo, Krasimira Aleksandrova, Antje Hebestreit, Alfonso Siani, Paola Russo

**Affiliations:** 1 Institute of Food Sciences, National Research Council, Via Roma 64, 83100 Avellino, Italy; 2 Department of Precision Medicine, School of Medicine and Surgery, University of Campania “Luigi Vanvitelli”, S. Andrea Delle Dame-Via L. De Crecchio 7, 80138 Naples, Italy; 3 Leibniz Institute for Prevention Research and Epidemiology—BIPS, Achterstraße 30, 28359 Bremen, Germany; 4 Faculty of Human and Health Sciences, University of Bremen, Grazerstraße 2, 28359 Bremen, Germany

**Keywords:** advanced glycation end products, urinary AGEs, inflammation, CRP, biomarkers, children, I.Family project

## Abstract

Advanced Glycation End Products (AGEs) have been positively correlated with inflammation in adults, while inconsistent evidence is available in children. We evaluated the association between urinary AGEs, measured by fluorescence spectroscopy, and biomarkers of subclinical inflammation in 676 healthy children/adolescents (age 11.8 ± 1.6 years, M ± SD) from the Italian cohort of the I.Family project. Urinary fluorescent AGEs were used as independent variable and high-sensitivity C-reactive protein (hs-CRP) was the primary outcome, while other biomarkers of inflammation were investigated as secondary outcomes. Participants with urinary AGEs above the median of the study population showed statistically significantly higher hs-CRP levels as compared to those below the median (hs-CRP 0.44 ± 1.1 vs. 0.24 ± 0.6 mg/dL, M ± SD *p* = 0.002). We found significant positive correlations between urinary AGEs and hs-CRP (*p* = 0.0001), IL-15 (*p* = 0.001), IP-10 (*p* = 0.006), and IL-1Ra (*p* = 0.001). At multiple regression analysis, urinary AGEs, age, and BMI Z-score were independent variables predicting hs-CRP levels. We demonstrated for the first time, in a large cohort of children and adolescents, that the measurement of fluorescent urinary AGEs may represent a simple, noninvasive, and rapid technique to evaluate the association between AGEs and biomarkers of inflammation. Our data support a role of AGEs as biomarkers of subclinical inflammation in otherwise healthy children and adolescents.

## 1. Introduction

Advanced glycation end products (AGEs) are a heterogeneous group of compounds resulting from a spontaneous non-enzymatic and non-selective reaction between reducing sugars and proteins, known as a Maillard reaction [1,2]. The two major sources of human exposure to AGEs are: endogenous AGEs, generated by abnormal glucose metabolism or as a byproduct of lipid peroxidation, and exogenous AGEs, present in foods. 

More than 20 different AGEs have been identified. They can be classified in different groups based on their chemical structures and ability to emit fluorescence [3,4]. A recent review proposed the following classification for the numerous AGEs identified in vivo and in vitro [3]: (1) Fluorescent and cross-linked (fluorescent/crosslinked); (2) Nonfluorescent and non-cross-linked (nonfluorescent/non-cross-linked); (3) Nonfluorescent protein cross-linked; (4) Fluorescent non-cross-linked. The majority of AGEs that have been identified so far are characterized by fluorescence in the area around an excitation wavelength of 370 nm and an emission of 440 nm, thus AGE-specific fluorescence has been widely used to detect accumulation of fluorescent AGEs, such as pentosidine, in body fluids and tissues [5,6,7,8,9]. 

Small amounts of AGEs are generated in vivo as a normal consequence of metabolism, and, in normal physiological conditions, the rapid turnover of intracellular proteins and the action of pathways for AGE detoxification protect the tissues from high AGEs accumulation and their subsequent chemical damage [2]. However, during aging and under pathologic conditions, these defense systems are unable to properly prevent AGEs production [10].

Whatever their origins, AGEs bind their cell receptor (RAGE), initiating a cascade of intracellular signals that induce the release of inflammatory cytokines and produce higher levels of reactive oxygen species (ROS) [11]. The activation of AGE–RAGE axis is currently considered the principal mechanism by which AGEs activate inflammatory processes [12]. Specifically, the AGE–RAGE interaction triggers the sustained activation of Nuclear Factor kappa B (NF-kB) which translocates from the cytoplasm to the nucleus, stimulating gene transcription of pro-inflammatory cytokines and activation of Mitogen-Activated Protein Kinase (MAPK) pathway, thus providing a positive feedback mechanism to amplify the inflammatory response [13,14,15,16,17]. Moreover, the binding of AGEs to RAGE increases the levels of ROS through activation of Nicotinamide Adenine Dinucleotide Phosphate (NADPH) oxidase and mitochondrial pathways; consequently, the activity of superoxide dismutase (SOD), catalase and, indirectly, other endogenous antioxidant defenses are decreased [18]. 

To date, there is consistent experimental and pre-clinical evidence of the adverse role of AGEs in several chronic inflammatory conditions, namely diabetic complications, cardiovascular, kidney, and neurodegenerative diseases [12,19,20,21]. Furthermore, there is growing evidence of the impact of AGEs on metabolic health and the onset of metabolic syndrome as well as obesity [22].

Although the molecular mechanisms by which AGEs are implicated in the pathogenesis of a wide range of diseases are not fully elucidated, AGEs have been proposed as a biomarker of inflammation [19]. Circulating AGEs correlate with indicators of inflammation and oxidative stress in obese adults with metabolic syndrome, but similar, albeit weaker, correlations were also evident in obese adults without metabolic syndrome [23], suggesting that circulating AGEs may reflect the presence of subclinical inflammation even in otherwise healthy adults. In children, the links between AGEs, and inflammation are not well defined, with pediatric studies showing contrasting results, probably due to different demographic and clinical characteristics, along with relatively small sample sizes [24,25,26,27,28].

According to a comprehensive list of inflammatory markers compiled by Calder et al. [29], the major inflammation signals being explored in human studies are, among others, CRP and cytokines, notably Interleukin 6 (IL-6) and Tumor necrosis factor alpha (TNF-α). In particular, CRP is a sensitive biomarker of inflammation with a proven record of clinical use and well-established protocols for its analysis and detection. Elevated CRP, for instance, has long been considered as a risk indicator of cardiovascular disease [30], and increase in CRP level is also observed in a number of metabolic disorders, including obesity [31]. Interestingly, we showed that healthy children with higher baseline levels of high-sensitivity C-Reactive Protein (hs-CRP) were at higher risk of developing overweight/obesity during growth [32]. Experimental evidence suggests that AGEs may be involved in the regulation of inflammatory markers, including CRP synthesis, via stimulation of IL-6 and IL-1b [33], or through the interaction of AGEs with their receptor RAGE [34,35]. 

Despite the accumulating evidence on the role of AGEs in diverse pathological conditions, there are no widely accepted analytical methods to determine the amounts of AGEs in body fluids [3]. Different methodologies for the analysis of AGEs were proposed, each of them presenting advantages and disadvantages. Among them, the relatively easy and inexpensive spectrofluorimetric assay allows the quantification of fluorescent AGEs in body fluids and would be useful as a screening test in population settings. In particular, it has been used to test the association between urinary AGEs and metabolic syndrome [36], and to discriminate urinary AGEs excretion over a wide range of kidney failure [37], thus suggesting the usefulness of this method as a rapid test for determining the association of AGEs with related pathological conditions. The non-invasive measurement of urinary fluorescent AGEs may be particularly appropriate for large scale studies in children and adolescent populations. 

The aim of the present study is to evaluate the association between urinary AGEs, measured by fluorescence spectroscopy and markers of subclinical inflammation [29] in children/adolescents of the Italian cohort of the I.Family study. The association of the urinary fluorescent-AGEs with hs-CRP was the primary outcome of the study, while the secondary outcomes included the association of urinary fluorescent-AGEs with other inflammatory markers. 

## 2. Materials and Methods

### 2.1. Experimental Design and Cohort

**Figure 1:**
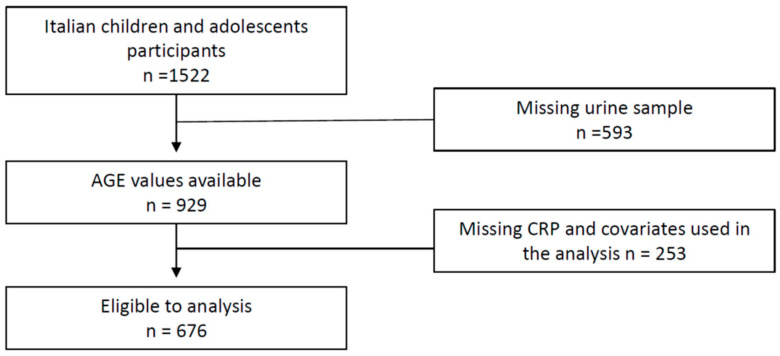
Flow chart of participants included in the final analysis.

The I.Family project, which aimed to assess the determinants of eating behavior in children and adolescents of eight European countries and related health outcomes, was built on the IDEFICS cohort, established in 2006 and followed up in 2012–2013 [38]. Briefly, the Italian cohort of the I.Family project was composed by 1522 children and teens who underwent a general examination module [38]. Among them, 929 participants provided a fasting urine sample for AGEs determination. Finally, after the exclusion of 253 participants with incomplete dataset, 676 cases were included in the present analysis. The flow chart of the selection process is shown in Figure 1. The study was conducted in accordance with the Declaration of Helsinki and approved by the Ethics Committee of the local Health Authority (ASL Avellino), and informed written parental consent was obtained for each participant.

Registration: The Pan-European IDEFICS/I.Family children cohort is registered under ISRCTN62310987. Date assigned: 23 February 2018.

### 2.2. Sample Processing and Analytical Procedures

A detailed description of sample collection and analytical procedures has been previously published [39]. The fasting venous blood was collected in BD Vacutainer ^®^ blood collection tubes according to standard operating procedures [39]. Samples were processed at the local survey centers and shipped to the central biorepository and to the clinical laboratory for analysis at regular intervals. At the first visit at the study center, a collection cup and instructions were given to the children and adolescents or their parents for the collection of morning urine samples. The morning urine was collected at home and brought to the study center on the same day (94% of the samples were first morning urine). No preservative was used, but parents were instructed to cool down the urine sample in the home fridge if the time span between collection and handing over at the study center exceeds two hours. At the study center, urine samples were stored at − 80 °C on the same day of collection.

### 2.3. Inflammatory Biomarkers

Inflammatory markers were measured by ELISA using electrochemiluminescent multiplex assay (using either single or MULTI-SPOT^®^ Assay Systems, Meso Scale Discovery-MSD, Rockville, MD, USA) on serum samples stored at −80 °C. A previous methodological study showed an overall good reliability of this method for the measurement of cytokines [40]. hs-CRP was measured with a single-plex assay, while Interferon gamma-induced protein 10 (IP-10), Interleukin 15 (IL-15), Interleukin 6 (IL-6), Interleukin 8 (IL-8), Tumor necrosis factor alpha (TNF-α), and interleukin-1 receptor antagonist (IL-1Ra) were run together on a 6-plex assay. The combination of biomarkers for the assays were decided based on the feasibility of combinations with the help of MSD customer support, as previously described [41]. 

### 2.4. Fluorescence

Urinary fluorescent-AGEs measurements were performed on a Perkin Elmer LifeSciences LS 55 spectrofluorimeter. Urine samples were diluted at 1:10 in phosphate-buffered saline, and fluorescence spectra were recorded between 400 nm and 600 nm, upon excitation at 370 nm, at room temperature. The fluorescence intensity was measured in correspondence of emission maximum centered at 440 nm and was corrected by subtracting the background. As the urinary AGEs concentration depends on the urine volume, the relative fluorescence intensity (expressed in arbitrary units, AU) was adjusted for the urinary creatinine concentration expressed as g/L. Urinary creatinine was measured by a colorimetric assay based on Jaffe’s reaction (COBAS INTEGRA 400 plus, Roche Diagnostics Ltd., CH-6343 Rotkreuz, Switzerland). 

### 2.5. Anthropometric and Blood Pressure Measurements

A detailed description of the anthropometric measurements in the I.Family project, including intra- and inter-observer reliability, has been published elsewhere [42]. Briefly, weight was determined to the nearest 0.1 kg using a body composition analyzer (Tanita BC 420 SMA, Tanita Europe GmbH, Sindelfingen, Germany) with participants in fasting status, without shoes and with light clothing. Height was measured with a calibrated stadiometer (Seca 225, Seca GmbH & Co., KG., Hamburg, Germany) and recorded to the nearest 0.1 cm. BMI was calculated by dividing body weight (in kg) by height squared (in m2). Age- and sex-specific BMI z-scores were calculated according to Cole and Lobstein [43].

Blood pressure measurement was performed during the day of the physical examination, in a quiet and warm room. Systolic BP (SBP) and diastolic BP (DBP) were measured at the right arm while the child was in a seated position with the back supported, uncrossed legs, feet on the floor, with the upper arm at the heart level. Children were asked to sit for at least 5 min before the measurement, and they were advised to avoid stimulant food/drinks and physical activity within the last 30 min before measurement. An automated oscillometric device (Welch Allyn 4200B-E2 Inc, Skaneateles Falls, NY, USA) [44] was used according to a standardized procedure. For the choice of the appropriate cuff size, arm circumference was measured using an inelastic tape (Seca 200, Birmingham, UK). Detailed procedures have been previously described [45]. Two measurements were taken with 2 min intervals, plus a further one in the case of a >5% difference in BP between the first two readings.

### 2.6. Medical History and Medication Use

Parents reported medication use and medical history for their children by means of an interview based on the health and lifestyle questionnaire. Parents were specifically asked to indicate whether their child had taken any kind of medication, including self-prescribed drugs, vitamins, and mineral supplements, during the week before blood sample drawing.

### 2.7. Statistical Analysis

In the descriptive analysis, urinary AGEs concentration was reported as median and interquartile range (IR). Normally distributed continuous variables were reported as Mean ± SD. Correlation analyses between continuous variables were performed using Spearman’s rho (ρ) for non-normally distributed data. The study population was stratified into two groups, using the median of urinary AGEs concentration as cut-points. The difference in hs-CRP between the two groups were tested with the Mann–Whitney U-test for non-normally distributed data. 

Univariate linear regression analysis was performed to study the association between urinary AGEs as dichotomous exposure variable, and hs-CRP as an outcome variable. hs-CRP was log-transformed to achieve normally distributed residuals. To identify possible confounders, we included in the regression variables that could theoretically affect hs-CRP, namely age (continuous exposure), sex (dichotomous exposure), and BMI Z-score (continuous exposure). IBM SPSS Statistics (Version 23.0. IBM Corp., Armonk, NY, USA) was used for the statistical analyses, and statistical significance was accepted at *p*-value less than 0.05. 

## 3. Results

In Table 1, the study population is presented according to the median of urinary AGEs. The median urinary AGEs concentration was 27,500 AU/g creatinine (interquartile range 11,700 AU/g creatinine). hs-CRP levels were significantly higher in participants with urinary AGEs concentration above the median (*p* = 0.002), while no significant differences between the two groups were observed with regard to sex distribution, age, anthropometric parameters and blood pressure.

Table 2 shows the correlations between urinary AGEs and parameters of inflammation in the entire study population. At univariate analysis, statistically significant and positive associations were observed between urinary AGEs and hs-CRP (*p* = 0.0001), IP-10 (*p* = 0.006), IL-15 (*p* = 0.001), and IL-1Ra (*p* = 0.001). 

At linear regression analysis, urinary AGEs, age, and BMI Z-score were predictors of hs-CRP levels, with urinary AGEs concentration above the median associated with higher hs-CRP levels (*p* = 0.003) (Table 3). 

## 4. Discussion

In the present paper, we report the findings of the largest analysis of the association between AGEs and biomarkers of inflammation in a population of free-living healthy children and adolescents. This study is, to our knowledge, the first to demonstrate that urinary AGE fluorescence intensity is positively associated with markers of sub-clinical inflammation in otherwise healthy children and adolescents. Although the contribution of AGEs to the inflammatory state has been typically observed in chronic, age-related inflammatory diseases [12], our findings suggest that there is potential for urinary fluorescent AGEs as early biomarkers of subclinical inflammation in children and adolescents. 

Only a few studies specifically evaluated the association of AGEs and markers of inflammation in children and adolescents. Accacha et al. examined the association of the AGE Nε-carboxymethyl-lysine (CML) with inflammatory markers in 88 middle school-age children, suggesting that, at variance with studies in adults, CML was negatively associated with IL-6 [25]. Heier et al. reported that serum methylglyoxal-derived hydroimidazolone-1 (MG-H1), an AGE compound present in serum and tissues, was positively associated with C-reactive protein in children and adolescents with diabetes [26]. Garay-Sevilla et al. did not find any correlation between serum CML and markers of inflammation, namely IL-6 and TNF-α in 80 normal weight and 80 obese Mexican adolescents [27]. Corica et al. showed that serum concentration of AGEs, measured by spectrofluorimetric detection, was positively associated with CRP in a pediatric cohort [28]. The relative discrepancy of the aforementioned findings may be attributable to different factors, including different demographic and clinical characteristics, relatively small sample sizes, and different methods used for the measurement of AGEs. Our choice to measure fluorescent AGEs in the urine was supported by previous evidence [36,37,46,47] and was dictated by the need to identify a non-invasive and non-expensive marker to be used in large scale population screening in pediatric populations. 

In 1998, Yanagisawa et al. measured AGE-specific fluorescence in the serum and urine of diabetic subjects [46]. They observed a significant correlation between serum and urinary concentration of fluorescent AGEs. There was a significant correlation between AGE–peptide levels measured by enzyme-linked immunosorbent assay (ELISA) and levels determined from the specific fluorescence intensity. De La Maza et al. used fluorescence spectroscopy to evaluate urinary excretion of AGEs in diabetic and non-diabetic elderly [47]. More recently, Suehiro et al. measured urinary AGEs using a fluorescence assay in 387 Japanese adults with or without metabolic syndrome [36]. Their findings suggest that the measurement of urinary fluorescent AGE levels may be useful as a simple test for the screening of metabolic syndrome [36]. In 2020, Steenbeke et al. aimed to investigate the possibilities of fluorescence spectroscopy to detect urinary AGEs in patients with different degrees of kidney insufficiency and in healthy controls [37]. They showed that UV fluorescence is a fast, simple, and affordable method for detecting urinary AGEs in patients with chronic kidney diseases. Interestingly, they also observed a positive correlation between the urinary AGE fluorescence intensity and the CRP levels, thus confirming the role of AGEs as markers of inflammation. 

Urinary excretion of AGEs may indeed reflect tissue AGEs accumulation and provide an estimate of the recent exposure of the body to AGEs [48]. Having in mind that no standardized method for the measurement of AGEs has been proposed so far [3], the estimation of fluorescent AGEs in urine represents a non-invasive method to be used for research purposes, and, in perspective, could be proposed for monitoring subclinical inflammation in population groups, apparently free of overt disease, like healthy children and adolescents. The discussion of the methods that have been developed to measure the concentration of AGEs in different human fluids is far beyond the scope of this paper and has been extensively reported elsewhere [3]. Briefly, the advantages of the measurement of fluorescent AGEs in urine are the relative simplicity and affordability as compared to other methods commonly used to measure AGEs such as ELISA, HPLC, and LC-MS/MS. On the other hand, this method also presents important drawbacks. For instance, it does not allow to identify the specific molecules responsible for the fluorescent signal, and it provides only the quantification of fluorescent AGEs, while in the urine non-fluorescent AGEs, CML, and MG-H1 are also present, thus possibly underestimating the amount of urinary AGEs [3].

A strength of the present study is the use of precisely standardized phenotypic measurements in study participants. In fact, all measurements were conducted according to standard operating procedures reported in detail elsewhere [49]. A limitation is the use of a single measurement of hs-CRP and urinary AGEs. However, the biological plausibility of the association between urinary AGEs excretion and inflammation is supported by the measurement of other inflammatory biomarkers, mainly confirming the direction of the association. It also needs to be acknowledged that a potential limitation of our study is its cross-sectional design, which does not allow the demonstration of any pathogenetic mechanism. 

## 5. Conclusions

The main discovery in this study is that urinary AGE fluorescence intensity is positively associated with markers of sub-clinical inflammation in otherwise healthy children and adolescents. This method could represent a simple alternative for detecting AGEs levels as biomarkers of different pathologies in epidemiological settings with respect to more sophisticated ones requiring trained personnel, laboratory facilities, and financial resources. However, the above-described methodological limitations of the fluorescent AGEs measurement should be kept in mind, and confirmation of the present findings with other quantification methods would be warranted.

## Figures and Tables

**Table 1:** Characteristics of the study population stratified by the median of urinary AGEs.

	Below Median (n 361)	Above Median (n 315)	*p*-Value
Urinary AGEs (AU)	230.4 ± 49.0	384.3 ± 123.0	0.0001
Sex (F/M %)	45.3/54.7	48.6/51.4	0.457
Age (years)	11.8 ± 1.6	11.8 ± 1.7	0.876
Height (cm)	151.3 ± 11.6	151.2 ± 11.2	0.232
Weight (kg)	50.5 ± 13.8	51.1 ± 15.0	0.578
BMI z-score	1.2 ± 1.0	1.3 ± 1.1	0.294
SBP (mm Hg)	108.0 ± 9.0	108.4 ± 9.7	0.565
DBP (mm Hg)	65.0 ± 6.4	65.4 ± 6.4	0.343
hs-CRP (mg/dL)	0.24 ± 0.6	0.44 ± 1.1	0.002 *

M ± SD; *: Mann–Whitney U-test for non-normally distributed data. Urinary AGEs: fluorescence intensity (expressed in arbitrary units, AU) adjusted for the urinary creatinine; BMI z-score: Age- and sex-specific BMI z-score, calculated according to Cole and Lobstein [43]; SBP: systolic blood pressure; DBP: diastolic blood pressure.

**Table 2:** Correlations between the urinary advanced glycation end products (AGE)-specific fluorescence intensity and the investigated inflammation parameters.

Inflammation Parameters	Urinary AGEs	
	Correlation Coefficient (Spearman’s Rho)	*p*-Value (2 Tailed)
hs-CRP	0.258	0.0001
IP-10	0.105	0.006
IL-15	0.131	0.001
IL-6	0.050	0.198
IL-8	−0.007	0.848
TNF-α	0.004	0.912
IL-1Ra	0.138	0.001

N = 676; Urinary AGEs: fluorescence intensity (expressed in arbitrary units, AU) adjusted for the urinary creatinine; hs-CRP: high sensitivity C-reactive protein; IP-10: Interferon gamma-induced protein 10; IL-15: Interleukin 15; IL-6: Interleukin 6; IL-8: Interleukin 8; TNF-α: Tumor necrosis factor alpha; IL-1Ra: interleukin-1 receptor antagonist.

**Table 3:** Linear regression analysis model with hsCRP concentration as a dependent variable.

Dependent Variable	Independent Variables	B (SE)	*p*-Value
hsCRP(Ln) mg/dl	Sex (m/f)	−0.023 (0.064)	0.719
	Age (years)	−0.050 (0.020)	0.011
	BMI z-score	0.175 (0.030)	0.0001
	Urinary AGEs (category)	0.189 (0.064)	0.003

SE: standard error; hsCRP(Ln): high sensitivity C-reactive protein (log transformed); BMI z-score: Age- and sex-specific BMI z-score, calculated according to Cole and Lobstein [43]; (category): category defined according to the median of fluorescence intensity (expressed in arbitrary units, AU) adjusted for the urinary creatinine.

## Data Availability

All data produced or analyzed during this study are included in this article.
